# New‐onset atrial fibrillation in a young patient detected by smartwatch

**DOI:** 10.1002/ccr3.2887

**Published:** 2020-04-23

**Authors:** Subhankar Samal, Namrata Singhania, Saurabh Bansal, Anuradha Sahoo

**Affiliations:** ^1^ Department of Hospital Medicine Ascension Columbia St. Mary’s Hospital Milwaukee WI USA; ^2^ Department of Hospital Medicine Mount Carmel East Hospital Columbus OH USA; ^3^ Department of Internal Medicine University of Illinois at Peoria Peoria IL USA; ^4^ Eversana Milwaukee WI USA

**Keywords:** atrial fibrillation, electrocardiogram, smartwatch, stroke

## Abstract

Atrial fibrillation (Afib) is associated with 15%‐25% of strokes. Wearable devices like Apple watch can generate an electrocardiogram and can increase detection of Afib in general population. Early diagnosis and treatment can decrease the risk of strokes.

## Graphical abstract

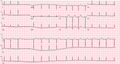

## 1 CASE DESCRIPTION

The use of wearable electronic devices like Apple watch and other smartwatches has been increasing. Optical sensors on wearable devices can detect irregular pulse. The process is known as photoplethysmography. We present a case of new‐onset atrial fibrillation detected by Apple watch in an otherwise healthy male.

A 39‐year‐old healthy male patient presented to hospital with palpitations for 4 hours. He did not have chest pain or dyspnea. His Apple watch series 3 showed variable heart rate between 60 and 130 per minute. He denied family history of heart problems. Serum electrolytes, complete blood count, TSH, and BNP were within normal limits. Urine analysis and drug screen were negative.

## 2 DIAGNOSIS

**FIGURE 1:**
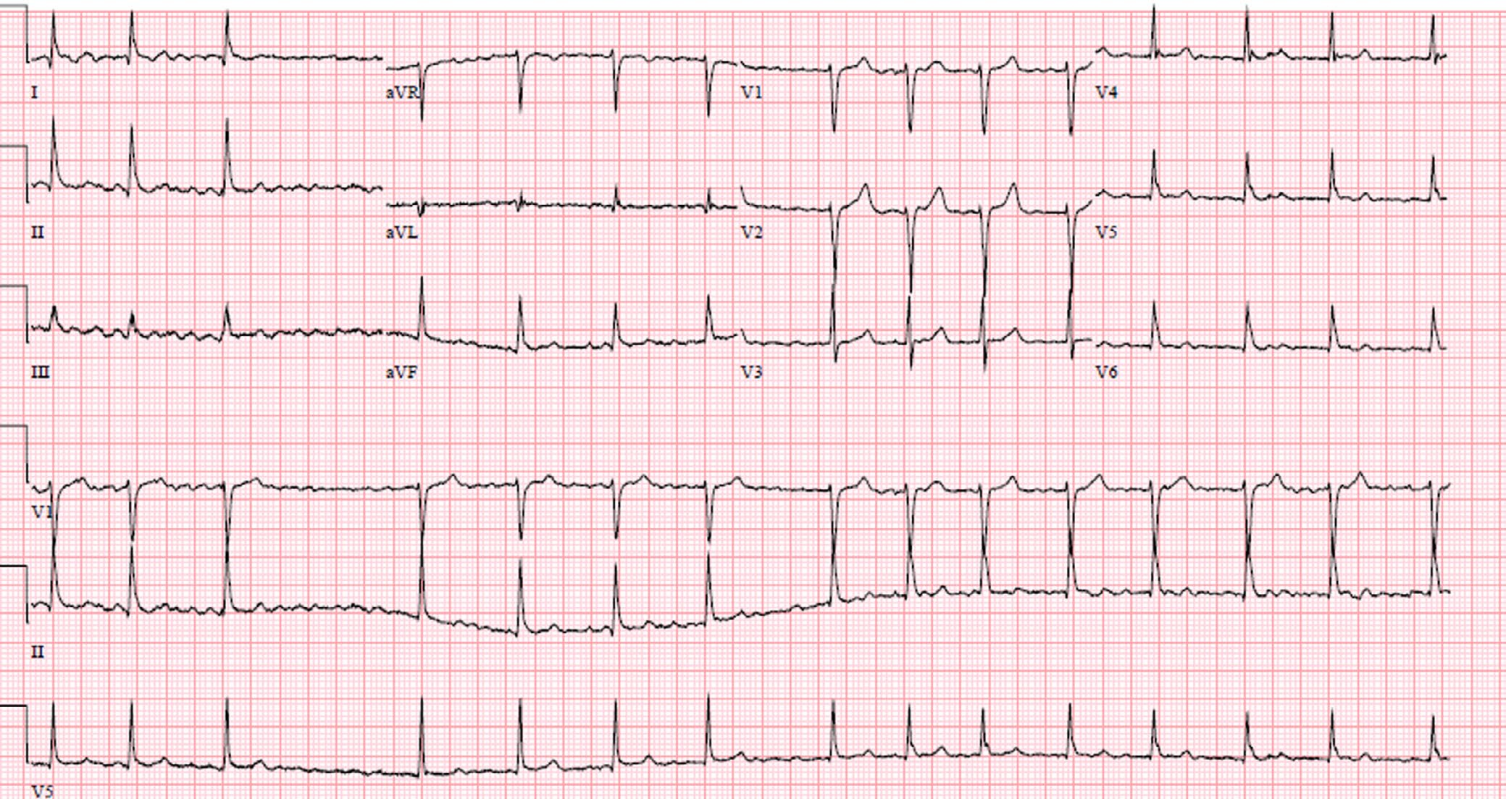
12‐lead electrocardiogram showing atrial fibrillation

12‐lead ECG showed atrial fibrillation (Afib; Figure 1). He was diagnosed with new‐onset Afib. Echocardiogram revealed 45% ejection fraction with no atrial thrombus. CHA2DS2‐VASc score was 1, and HAS‐BLED score was 0. He was chemically cardioverted using intravenous flecainide per cardiology recommendations. He maintained sinus rhythm afterward and was discharged home on metoprolol and apixaban. Smartwatches are creating a new era of monitoring personal cardiovascular metrics in real time by utilizing photoplethysmography.^1^ The ECG application on Apple watch series 4 can generate an ECG like a Lead 1ECG. The application demonstrated 99.6% specificity for sinus rhythm and 98.3% sensitivity for Afib classification for classifiable results.^2^ Ability of this algorithm to identify Afib has received FDA clearance. This potential to discover undiagnosed Afib in general population will have huge impact and can significantly decrease the risk of strokes if treated promptly.

## CONFLICT OF INTEREST

Authors do not have any conflict of interest to disclose.

## AUTHOR CONTRIBUTIONS

SS: prepared the manuscript and reviewed the literature. NS, SB, and AS: reviewed the literature and revised the manuscript.
